# Sleep characteristics of middle-aged adults with non-alcoholic fatty liver disease: findings from the Shahrekord PERSIAN cohort study

**DOI:** 10.1186/s12889-023-15251-4

**Published:** 2023-02-11

**Authors:** Elaheh Zarean, Mehdi Azizmohammad Looha, Payam Amini, Ali Ahmadi, Pierre-Antoine Dugué

**Affiliations:** 1 Modeling in Health Research Center, Shahrekord University of Medical Sciences, Shahrekord, Iran; 2 Precision Medicine, School of Clinical Sciences at Monash Health, Monash University, Clayton, VIC Australia; 3 Department of Biostatistics, Faculty of Paramedical Sciences, Shahid Beheshti University of Medical Science, Tehran, Iran; 4 Department of Biostatistics, School of Public Health, IRAN University of Medical Sciences, Tehran, Iran; 5 Department of Epidemiology and Biostatistics, School of Health and, Modeling in Health Research Center, Shahrekord University of Medical Sciences, Shahrekord, Iran; 6 Cancer Epidemiology Division, Cancer Council Victoria, Melbourne, VIC Australia; 7 Centre for Epidemiology and Biostatistics, Melbourne School of Population and Global Health, The University of Melbourne, Parkville, VIC Australia

**Keywords:** Sleep duration, Sleep quality, Fatty liver disease, Cohort study, Quality of life

## Abstract

**Background:**

Several studies have reported short sleep duration in people with non-alcoholic fatty liver disease (NAFLD) but other sleep characteristics have been less studied. We aimed to assess the cross-sectional association of NAFLD with sleep duration and quality in an Iranian population sample.

**Methods:**

We used data from 9,151 participants in the Shahrekord Prospective Epidemiological Research Studies in Iran (PERSIAN) Cohort Study, including 1,320 that were diagnosed with NAFLD. Log-binomial regression models sequentially adjusted for sociodemographic, lifestyle, clinical and biological variables were used to estimate relative risks (RR) and 95% confidence intervals (95% CI) for the association between NAFLD and sleep characteristics.

**Results:**

Participants with NAFLD had shorter sleep duration, later wake-up time and bedtime, worse sleep efficiency, and more frequent daytime napping and use of sleeping pills, in age- and sex-adjusted models. After controlling for sociodemographic, lifestyle, clinical, and biological variables the associations remained strong for sleep efficiency (per 10%, RR = 0.92, 95%CI: 0.88–0.96) and use of sleeping pills (RR = 1.48, 95%CI: 1.17–1.88). The association between NAFLD and sleep efficiency was stronger in participants aged > 60 years (RR = 0.81, 0.70–0.93) and 40–60 years (RR = 0.87, 0.82–0.94), compared with those aged < 40 years (P-heterogeneity < 0.001). More frequent daytime napping in participants with NAFLD, compared with non-NAFLD, was observed in males but not females (P-heterogeneity = 0.007), and in those with body mass index (BMI) < 30 but not in obese participants (P-heterogeneity < 0.001).

**Conclusions:**

Diagnosis of NAFLD is associated with several poor sleep characteristics in middle-aged Iranians. Although longitudinal studies would help to clarify the direction of causality, our study shows that poor sleep is an important aspect of NAFLD.

## Background

Non-alcoholic fatty liver disease (NAFLD) is one of the most prevalent liver diseases worldwide and may lead to serious complications such as non-alcoholic steatohepatitis, cirrhosis, end-stage liver disease and liver cancer [1, 2]. NAFLD is characterized by the excessive accumulation of hepatic fat, with the presence of steatosis in > 5% of hepatocytes, assessed by magnetic resonance imaging or histology image analysis of liver proton density fat fraction [2]. This chronic liver disease is recognized as the hepatic manifestation of obesity and metabolic syndrome (MetS). MetS is defined as having three or more of excess central obesity, raised blood pressure, high levels of triglycerides, low levels of high-density lipoproteins and impaired fasting glucose or diabetes, and is thought to be both a cause and a consequence of NAFLD [3]. Several other non-metabolic factors play a role in NAFLD including lifestyle factors such as low levels physical activity and poor dietary habits [4, 5]

Sleep disturbances are known to be associated with insulin resistance and dysregulation of glucose metabolism, which may lead to metabolic disorders such as type 2 diabetes (T2D), obesity, and MetS [6–15]. Two recent meta-analyses investigated sleep issues in NAFLD patients but these included only a handful of studies and focused exclusively on sleep duration [9]

To the best of our knowledge, other sleep characteristics have rarely been studied. A case–control study in South Korea suggested that NAFLD patients were more likely to suffer from obstructive sleep apnea, particularly in those with excessive daytime sleepiness [16]. In Chinese middle-aged and elderly adults, participants with longer daytime napping and shorter sleep duration were more frequently diagnosed with NAFLD [17]. The large cross-sectional study by Kim et al. found that both poor sleep quality and short duration were associated with NAFLD [18].

Given the strong interplay between NAFLD and other metabolic conditions, thorough adjustment for potential confounding variables when analyzing sleep patterns in relation to NAFLD patients is paramount, and this aspect may have been neglected in some of the previous studies. We hypothesized that people with NAFLD have worse sleep quality, independently of their other lifestyle and clinical characteristics. To our knowledge, few studies have assessed the sleep characteristics of NAFLD patients, particularly in the Middle Eastern population, and these only focused on specific sleep variables (mainly duration). The aim of this study was to clarify the cross-sectional relationship between NAFLD diagnosis and sleep characteristics in an Iranian population sample.

## Methods

### The Shahrekord cohort study

This cross-sectional study was conducted using data collected from participants in the prospective Shahrekord Cohort Study (SCS), a population-based study of 10,075 men and women aged 35 to 70 years. The SCS is one of the Prospective Epidemiological Research Studies in Iran (PERSIAN) [19] and recruited people from Chaharmahal and Bakhtiari province, located in the southwest of Iran. The SCS was designed as a regional cohort study in Shahrekord and is planned to have a 20-year follow-up. Enrolment in the SCS started in November 2015 and ended in June 2018. More details about the PERSIAN cohorts and the SCS protocol can be found in other publications [20, 21]. The SCS protocol was approved by the Ethics Committee of Shahrekord University of Medical Sciences (IR.SKUMS.REC 1394.286).

### Study sample

We excluded from the study: pregnant women (*n* = 18), participants with alcoholic liver disease, or who consumed a large amount of alcohol (more than 20 g daily for women and 30 g daily for men) and those with grade III NAFLD (*N* = 52), and those with ischemic heart disease (*N* = 554), myocardial infarction (*N* = 123), hepatitis B and hepatitis C (*N* = 49), and cancer (*N* = 71). No participants with viral or autoimmune hepatitis or cognitive disorders were recruited in this study. After exclusions, 9,151 participants were available for the analysis.

### Sociodemographic and lifestyle variables

Sociodemographic variables including ethnicity group, educational attainment, marital status and socioeconomic status were collected using the SCS general questionnaire. Lifestyle variables such as smoking habits and alcohol and drug consumptions were collected using medical questionnaires. Physical activity was recorded using the general questionnaire and self-reported daily activities were converted to metabolic equivalent of tasks (METs).

### Anthropometric and clinical variables

The anthropometric characteristics of the participants were measured using a wall height meter (Seca 206) for height, an analog scale (Seca) for weight, and standard tape for waist, hip, and wrist circumferences. Blood pressure at the left and right arm was measured twice at a 15-min interval using a standard barometer (Richter), and the average of these two measures was calculated. A Pars Azmoon kit was used to measure the following biochemical variables in blood serum of all participants: fasting blood glucose, serum cholesterol (total, high-density lipoprotein [HDL], low-density lipoprotein [LDL]), alanine aminotransferase (ALT), aspartate aminotransferase (AST), and triglycerides (TG). MetS was diagnosed using the National Cholesterol Education Program Adult Treatment Panel III criteria (ATPIII) definition (13), i.e. meeting any three of the following criteria: fasting serum glucose ≥ 100 mg/dl, waist circumference (WC) (> 102 cm in men; > 88 cm in women), TG ≥ 150 mg/dl, HDL (< 40 mg/dl in men; < 50 mg/dl in women), systolic blood pressure (SBP) ≥ 140 or diastolic blood pressure (DBP) ≥ 90 mmHg (25).

### NAFLD diagnosis

NAFLD status was determined using liver ultrasound imaging, which was retrieved from the medical records of participants and checked by a clinician [22].

### Sleep characteristics

Sleep characteristics were assessed with the validated 9-item sleep and circadian rhythm questionnaire used in all PERSIAN cohorts [19]. The variables retained for this study were sleep duration (hours), sleep efficiency (%), bedtime, daytime napping (hours), falling asleep unintentionally, presence of leg restless syndrome, and use of sleeping pills. Total time in bed was calculated as the difference between the times at which participants reported they went to bed at night and left their bed in the morning. Sleep efficiency was calculated as the ratio of sleep time duration to total time in bed multiplied by 100 [23].

### Statistical analysis

Continuous variables were presented as their means and standard deviations (SD) and categorical variables as numbers and percentages [24]. Although logistic regression is typically used when the outcome of interest is binary, the odds ratio is not a good estimate of the relative risk when this outcome is not rare; the log-binomial model is a reliable alternative to estimate the relative risk [25, 26]. In this study, as the prevalence of NAFLD was not rare (14.4%), log-binomial regression was used to estimate relative risks (RR) and 95% confidence intervals (95% CI) for the association between sleep characteristics and NAFLD status. Associations were first evaluated using Model 0 adjusting for age and sex, and then using four multivariable models sequentially adjusted for potential confounders: Model 1 adjusted for sociodemographic variables including age, sex, ethnicity, marital status, education, and socioeconomic status; Model 2 adjusted for variables in Model 1 and lifestyle variables including physical activity, smoking status, alcohol consumption, and use of drugs; Model 3 adjusted for the variables in Model 2 along with clinical variables (T2D, hypertension and BMI); Model 4 adjusted for the variables in Model 3 and biological markers of metabolic disturbances (TG, HDL, and LDL).

Heterogeneity in the associations by age, sex, and BMI was tested by comparing models (Model 2) with and without interaction terms of these factors with the sleep variables using likelihood ratio tests. The participants’ characteristics were described in the presence of missing values. All other analyses were performed using participants without missing values in any of the models (96%, *N* = 8,790).

All statistical analyses were conducted using R software version 3.6.3.

## Results

### Baseline characteristics of participants

Of the 9,151 participants included in the study, 4,211 (46.4%) were male, their mean ± SD age was 49.1 ± 9.2, and 1,320 participants (479 men and 841 women) were diagnosed with NAFLD (Table 1). Compared with non-NAFLD participants, those with NAFLD had strongly elevated fatty liver index (64.9 vs 46.9) and hepatic steatosis index (43.7 vs 36.9), as well as on average higher BMI (mean ± SD: 29.9 ± 4.4), SBP (117.6 ± 16.2), DBP (77.3 ± 10.5), and metabolic variables including total cholesterol (191 ± 40), triglycerides (174 ± 97), fasting glucose (102 ± 31), AST (21 ± 10), and ALT (26 ± 17). The NAFLD group also had higher prevalence of T2D (29%), hypertension (25%) and metabolic syndrome (33%) than the non-NAFLD group (13%, 13%, and 19%, respectively) (Table 1).

**Table 1:** Baseline characteristics of participants in the Shahrekord cohort study (*n* = 9,151)

Variables	NAFLD	Non-NAFLD	*P*
	(*N* = 1,320)	(*N* = 7,831)	
	n (%)/ mean ± SD	n (%)/ mean ± SD	
Demographic variables			
Age, year	50 ± 9	49 ± 9	< 0.001
Sex			< 0.001
Male	479 (36.3)	3732 (47.7)	
Female	841 (63.7)	4099 (52.3)	
Ethnicity			< 0.001
Fars	624 (47.3)	3043 (38.9)	
Turk	108 (8.2)	512 (6.5)	
Lur Bakhtiari	521 (39.5)	3966 (50.6)	
Others	67 (5.1)	310 (4)	
Education			< 0.001
Illiterate	403 (30.5)	2560 (32.7)	
< Diploma degree	529 (40.1)	3422 (43.7)	
≥ Diploma degree	380 (28.9)	1789 (22.8)	
Marital status			< 0.001
Single	12 (0.9)	143 (1.8)	
Married	1253 (94.9)	7343 (93.8)	
Divorced	49 (3.7)	285 (3.6)	
Widowed	6 (0.5)	60 (0.8)	
^a^ Socioeconomic status			< 0.001
Low	330 (25)	2664 (34)	
Medium	459 (34.8)	2616 (33.4)	
High	519 (39.3)	2467 (31.5)	
Anthropometric characteristics and metabolic parameters			
Weight, Kg	78.6 ± 13.4	72.3 ± 13.1	< 0.001
BMI, Kg/m2	29.9 ± 4.4	27.1 ± 4.5	< 0.001
Waist circumference, cm	99.4 ± 10.5	93.7 ± 11.3	< 0.001
Hip circumference, cm	103.9 ± 8.1	100.5 ± 7.7	< 0.001
Wrist circumference, cm	17.6 ± 1.5	17.3 ± 1.4	< 0.001
Total cholesterol, mg/dL	191 ± 40	184 ± 41	< 0.001
HDL cholesterol, mg/dL	51.1 ± 11.4	50.7 ± 11.6	0.31
LDL cholesterol, mg/dL	105 ± 33	104 ± 33	0.44
TG, mg/dL	174 ± 97	145 ± 85	< 0.001
Fasting glucose, mg/dL	102 ± 31	95 ± 25	< 0.001
AST, IU/L	21 ± 10	19 ± 8	< 0.001
ALT, IU/L	26 ± 17	21 ± 13	< 0.001
SBP, mmHg	117.6 ± 16.2	114.4 ± 17.2	< 0.001
DBP, mmHg	77.3 ± 10.5	74.9 ± 10.5	< 0.001
Life style variables			
Physical Activity, METs	39.2 ± 6.4	41.4 ± 8.9	< 0.001
Smoking (yes)	158 (12)	1232 (15.7)	< 0.001
Alcohol Consumption (yes)	196 (14.8)	1263 (16.1)	0.13
Using drug (yes)	151 (11.4)	1269 (16.2)	< 0.001
Medical history and NAFLD severity assessments			
T2D (yes)	235 (28.8)	582 (13.4)	< 0.001
Hypertension (yes)	323 (24.5)	1047 (13.4)	< 0.001
MetS (yes)	436 (33)	1487 (19)	< 0.001
FLI	64.9 ± 23.2	46.9 ± 27.6	< 0.001
HSI	43.7 ± 13.1	36.9 ± 9.0	< 0.001
Sleeping characteristics			
Sleeping time duration, hour	6.8 ± 1.5	6.9 ± 1.6	0.08
Sleeping time duration category, hour			0.11
≤ 7	825 (62.5)	4669 (59.6)	
7–9	379 (28.7)	2373 (30.3)	
≥ 9	116 (8.8)	788 (10.1)	
Bedtime duration, hour	7.3 ± 1.5	7.3 ± 1.5	0.94
Bed time, hour,			< 0.001
≤ 10 p.m	263 (19.9)	2046 (26.1)	
10 p.m.-12 a.m	559 (42.3)	3260 (41.6)	
≥ 12 a.m	498 (37.7)	2524 (32.2)	
Wake up time, hour			0.003
≤ 6 a.m	617 (46.7)	4035 (51.5)	
6–9 a.m	612 (46.4)	3363 (42.9)	
≥ 9 a.m	91 (6.9)	433 (5.5)	
Daytime nap (yes)	796 (60.3)	4206 (53.7)	< 0.001
Daytime nap, hour	0.95 ± 0.6	0.96 ± 0.6	0.96
Sleep Efficiency (%)	92.3 ± 16.3	95.0 ± 34.3	< 0.001
Falling asleep unintentionally (yes)	285 (21.6)	1508 (19.3)	0.05
Using sleeping pills (yes)	117 (8.9)	374 (4.8)	< 0.001
Restless legs syndrome (yes)	195 (14.8)	916 (11.7)	0.002

Data are presented as mean (SD) for continuous, and n (%) for categorical variables. *P*-values reported for t-test and chi-square test

Abbreviations: *SD* standard deviation, *T2D* type 2 diabetes, *MetS* metabolic syndrome, *HIS* Hepatic Steatosis Index, *FLI* Fatty Liver Index, *SBP* systolic blood pressure, *DBP* diastolic blood pressure, *AST* aspartate aminotransferase, *ALT* alanine transaminase, *HDL* high-density lipoprotein, *LDL* low-density lipoprotein, *TG* triglycerides, *NAFLD* non-alcoholic fatty liver disease

^**a**^ Socioeconomic status was obtained by measuring several variables relating to income, occupation, and educational attainment. Principal component factor analysis was applied to these variables, and the first principal component was taken as a measure of socioeconomic status, and transformed to a three-category ordinal variable (low / medium / high) using its tertiles

### Association between sleep characteristics and NAFLD

In age- and sex-adjusted models (Model 0), several sleep characteristics were negatively associated with NAFLD: sleep duration (per one hour, RR = 0.95, 95%CI: 0.92–0.99), waking up earlier than 6a.m. (RR = 0.76, 95%CI: 0.62–0.94, compared with ≥ 9a.m.), bedtime hour ≤ 10p.m. compared with ≥ 12a.m. (RR = 0.65, 95%CI: 0.56–0.74), bedtime hour 10p.m.-12a.m. compared with ≥ 12a.m. (RR = 0.88, 95%CI: 0.79–0.99), sleep efficiency (per 10%, RR = 0.93, 95%CI: 0.89–0.97), and reporting restless legs syndrome (RR = 0.81, 95%CI: 0.71–0.93). Participants were more likely to have NAFLD when they reported daytime napping (RR = 1.29, 95%CI: 1.17–1.44) and using sleeping pills (RR = 1.57, 95%CI: 1.33–1.86) (Table 2).

**Table 2:** Association between NAFLD status and sleep characteristics using log-binomial models (*N* = 1320 cases of NAFLD; 7831 non-cases)

Sleep characteristics	Model 0^a^		Adjusted models							
			**Model 1**^**b**^		**Model 2**^**c**^		**Model 3**^**d**^		**Model 4**^**e**^	
	RR^*^ (95% CI)	***P***	RR^*^ (95% CI)	***P***	**RR (95% CI)**	***P***	**RR (95% CI)**	***P***	**RR (95% CI)**	***P***
**Sleep duration (hour)**	0.95 (0.92,0.99)	0.03	0.98 (0.95,1.02)	0.31	0.97 (0.93,1.00)	0.05	0.99 (0.96,1.03)	0.63	0.98 (0.94,1.03)	0.41
**Sleep duration category (hour)**										
** ≤ 7**	1.25 (1.05,1.51)	0.04	1.08 (0.90,1.30)	0.40	1.20 (0.99,1.44)	0.06	1.06 (0.88,1.27)	0.54	1.11 (0.88,1.40)	0.36
** 7–9**	1.14 (0.94,1.39)	0.47	1.04 (0.86,1.27)	0.68	1.12 (0.92,1.36)	0.27	1.03 (0.85,1.24)	0.79	1.08 (0.85,1.38)	0.55
** ≥ 9**	Ref		Ref		Ref		Ref		Ref	
**Wake-up time category (hour)**										
** ≤ 6 a.m**	0.76 (0.62,0.94)	0.01	0.82 (0.67,1.00)	0.06	0.93 (0.75,1.14)	0.47	0.93 (0.77,1.13)	0.49	0.99 (0.75,1.29)	0.92
** 6–9 a.m**	0.91 (0.74,1.11)	0.30	0.93 (0.76,1.14)	0.51	1.01 (0.82,1.24)	0.95	1.00 (0.83,1.21)	0.98	1.06 (0.81,1.38)	0.69
** ≥ 9 a.m**	Ref		Ref		Ref		Ref		Ref	
**Bedtime category (hour)**										
** ≤ 10 p.m**	0.65 (0.56,0.74)	< 0.001	0.79 (0.68,0.91)	0.002	0.80 (0.69,0.93)	0.004	0.92 (0.79,1.06)	0.23	0.92 (0.77,1.10)	0.36
** 10 p.m.-12 a.m**	0.88 (0.79,0.99)	0.04	0.91 (0.82,1.02)	0.11	0.91 (0.82,1.02)	0.12	0.99 (0.89,1.10)	0.89	0.99 (0.86,1.14)	0.85
** ≥ 12 a.m**	Ref		Ref		Ref		Ref		Ref	
**Bedtime duration (hour)**	0.98 (0.94,1.01)	0.73	1.01 (0.98,1.05)	0.49	0.99 (0.95,1.02)	0.45	1.01 (0.98,1.05)	0.43	1.01 (0.96,1.06)	0.70
**Sleep efficiency (10%)**	0.93 (0.89,0.97)	< 0.001	0.92 (0.88,0.96)	< 0.001	0.92 (0.88,0.97)	0.001	0.95 (0.92,0.99)	0.005	0.92 (0.88,0.98)	0.009
**Daytime napping (hours)**	1.04 (0.96,1.12)	0.48	1.03 (0.95,1.12)	0.43	1.03 (0.95,1.11)	0.53	1.03 (0.95,1.11)	0.49	1.04 (0.94,1.15)	0.44
**Daytime napping (yes/no)**	1.29 (1.17,1.44)	< 0.001	1.18 (1.06,1.31)	0.002	1.12 (1.01,1.24)	0.03	1.03 (0.93,1.14)	0.60	1.07 (0.94,1.21)	0.33
**Falling asleep unintentionally (yes/no)**	1.10 (0.97,1.24)	0.56	1.09 (0.97,1.24)	0.15	1.08 (0.96,1.23)	0.20	1.05 (0.93,1.18)	0.43	1.04 (0.89,1.22)	0.60
**Restless legs syndrome (yes/no)**	0.81 (0.71,0.93)	0.001	0.78 (0.66,0.92)	0.004	0.78 (0.66,0.93)	0.005	0.85 (0.71,1.02)	0.07	0.87 (0.73,1.04)	0.13
**Using sleeping pills (yes/no)**	1.57 (1.33,1.86)	< 0.001	1.52 (1.28,1.79)	< 0.001	1.47 (1.24,1.74)	< 0.001	1.53 (1.20,1.93)	0.02	1.48 (1.17,1.88)	0.001

Abbreviations: Risk Ratios and 95% confidence intervals (RR [95% CI]) for estimated using log-binomial regression, *Ref.* Reference category

^a^ Model 0: adjusted on a sleeping characteristic, age and sex

^b^ Model 1: adjusted on a sleeping characteristic variable and demographic variables (sex, age, education, marital status, ethnicity, socioeconomic status)

^c^ Model 2: Model 1 + lifestyle variables (physical activity, smoking, alcohol consumption, use of drugs)

^d^ Model 3: Model 1 + Model 2 + clinical variables (hypertension, T2D, BMI)

^e^ Model 4: Model 1 + Model 2 + Model3 + biological variables (LDL, HDL, TG)

Note: Sex, age, marital status, physical activity, hypertension, T2D, BMI and triglycerides were associated with NAFLD in all multivariable log-binomial models

Note: BMI and Age were used as continuous variables in multivariable log-binomial models. Note: *P*-values were calculated using log-binomial regression

As shown in Table 2, adjustment for sociodemographic variables (Model 1) moderately attenuated the association observed for waking up earlier than 6a.m. (RR = 0.82, 95%CI: 0.67–1.00), going to bed earlier than 10p.m. (RR = 0.79, 95%CI: 0.68–0.91), use of sleeping pills (RR = 1.52, 95%CI: 1.28–1.79), daytime napping (RR = 1.18, 95%CI: 1.06–1.31), sleep efficiency (RR = 0.92, 95%CI: 0.88–0.96), and restless legs syndrome (RR = 0.78, 95%CI: 0.66–0.92). Additional adjustment for physical activity, alcohol consumption, smoking and use of drugs (Model 2) also left the associations virtually unchanged, compared with Model 1, except for waking up earlier than 6a.m. (point RR from 0.82 to 0.93) and daytime napping (point RR from 1.18 to 1.12) (Table 2).

Adjustment for key clinical variables (Model 3, Table 2) resulted in null associations for bedtime hour ≤ 10p.m., compared with ≥ 12a.m., sleep duration and daytime napping. The associations nevertheless remained quite strong for sleep efficiency and use of sleeping pills. In the comprehensively adjusted Model 4, participants with better sleep efficiency were less likely to have NAFLD (per 10%, RR = 0.92, 95%CI: 0.88–0.98). Participants using sleeping pills were 48% more likely to have a NAFLD diagnosis (RR = 1.48, 95%CI: 1.17–1.88) (Table 2).

### Effect modification by age, sex, and BMI

Results stratified by age, sex, and BMI are shown in Table 3. Most associations appeared consistent across subgroups, in particular for restless legs syndrome, use of sleeping pills, and waking-up time. For sleep efficiency, the associations varied by age, being substantially stronger in older participants (> 60 years: RR = 0.81, 95%CI: 0.70–0.93; ≤ 40 years: RR = 1.01, 95%CI: 0.97–1.04; P-interaction < 0.001), and by BMI, being stronger in obese participants (BMI ≥ 30: RR = 0.89, 95%CI: 0.84–0.94; BMI ≤ 25: RR = 1.01, 95%CI: 0.98–1.04; P-heterogeneity = 0.01). Male participants who reported daytime napping were 27% more likely to have NAFLD (RR = 1.27, 95%CI: 1.05–1.53) but no association was observed in females (RR = 0.94, 95%CI: 0.83–1.06), P-heterogeneity = 0.007. Daytime napping participants with a BMI ≥ 30 were less likely to have NAFLD (RR = 0.81, 95%CI: 0.70–0.94), but the opposite was found in those with BMI ≤ 25 and between 25 and 30 (RR = 1.24 and 1.29, respectively, P-heterogeneity < 0.001), Table 3. Weak evidence of effect modification by sex and age was also observed for the bedtime hour variable (P-interaction = 0.03 and 0.02, respectively); male participants with early bedtime were less likely to have NAFLD than those with late bed time (RR = 0.73, 95%CI: 0.58–0.93), but the same was not observed in females (RR = 0.99, 95%CI: 0.85–1.15). Older participants (age ≥ 60) with early bedtime were substantially less likely to have NAFLD (RR = 0.66, 95%CI: 0.49–0.90), but the same was not observed in participants aged < 60.

**Table 3:** Interaction between sleeping characteristics and the factors in association with NAFLD using likelihood ratio test

Sleep characteristics	Modifier	N	N events	RR	95% CI	*p*-interaction ^a^
**Sleep duration**	Male	-	-	0.97	0.92–1.03	0.78
	Female	-	-	0.98	0.94–1.03	
	Age ≤ 40	-	-	0.99	0.89–1.10	0.25
	Age 40–60	-	-	0.99	0.95–1.03	
	Age ≥ 60	-	-	0.92	0.84–1.00	
	BMI ≤ 25	-	-	0.91	0.83–1.00	0.17
	BMI 25–30	-	-	0.96	0.89–1.02	
	BMI ≥ 30	-	-	1.01	0.95–1.08	
**Wake up time category (hour) (≤ 6 a.m.)**	Male	-	-	0.85	0.71–1.01	0.20
	Female	-	-	0.98	0.86–1.11	
	Age ≤ 40	-	-	0.94	0.69–1.29	0.23
	Age 40–60	-	-	0.97	0.86–1.09	
	Age ≥ 60	-	-	0.75	0.58–0.98	
	BMI ≤ 25	-	-	1.08	0.78–1.48	0.62
	BMI 25–30	-	-	0.93	0.80–1.08	
	BMI ≥ 30	-	-	0.90	0.78–1.05	
**Bed time category (hour) (≤ 10 p.m.)**	Male	-	-	0.73	0.58–0.93	0.03
	Female	-	-	0.99	0.85–1.15	
	Age ≤ 40	-	-	1.27	0.88–1.83	0.02
	Age 40–60	-	-	0.93	0.80–1.09	
	Age ≥ 60	-	-	0.66	0.49–0.90	
	BMI ≤ 25	-	-	0.75	0.50–1.11	0.59
	BMI 25–30	-	-	0.92	0.75–1.12	
	BMI ≥ 30	-	-	0.92	0.77–1.12	
**Sleep efficiency**	Male	-	-	0.89	0.83–0.96	0.26
	Female	-	-	0.94	0.89–0.99	
	Age ≤ 40	-	-	1.01	0.97–1.04	< 0.001
	Age 40–60	-	-	0.87	0.82–0.94	
	Age ≥ 60	-	-	0.81	0.70–0.93	
	BMI ≤ 25	-	-	1.01	0.98–1.04	0.01
	BMI 25–30	-	-	0.92	0.86–0.99	
	BMI ≥ 30	-	-	0.89	0.84–0.94	
**Day time nap (yes)**	Male	2622	470	1.27	1.05–1.53	0.007
	Female	2380	326	0.94	0.83–1.06	
	Age ≤ 40	910	104	1.06	0.79–1.42	0.87
	Age 40–60	3369	579	1.02	0.90–1.15	
	Age ≥ 60	723	113	1.10	0.84–1.43	
	BMI ≤ 25	1290	85	1.24	0.90–1.71	< 0.001
	BMI 25–30	2297	396	1.29	1.10–1.52	
	BMI ≥ 30	1413	315	0.81	0.70–0.94	
**Restless legs syndrome**	Male	652	142	0.96	0.74–1.25	0.29
	Female	459	53	0.81	0.69–0.95	
	Age ≤ 40	162	16	1.02	0.62–1.68	0.71
	Age 40–60	770	150	0.83	0.71–0.97	
	Age ≥ 60	179	29	0.87	0.61–0.94	
	BMI ≤ 25	282	15	1.11	0.65–1.90	0.53
	BMI 25–30	490	89	0.81	0.66–0.99	
	BMI ≥ 30	339	91	0.85	0.70–1.03	
**Use of sleeping pills**	Male	340	93	1.52	0.95–2.43	0.60
	Female	151	24	1.75	1.35–2.29	
	Age ≤ 40	53	9	1.74	0.81–3.76	0.98
	Age 40–60	237	83	1.71	1.30–2.25	
	Age ≥ 60	111	25	1.63	0.99–2.69	
	BMI ≤ 25	124	11	1.43	0.73–2.81	0.86
	BMI 25–30	212	50	1.72	1.23–2.42	
	BMI ≥ 30	155	56	1.75	1.23–2.49	

Abbreviations: Risk Ratios and 95% confidence intervals (RR [95% CI]) for estimation using log-binomial regression

^a^ Interaction analyses were assessed using Model2 + BMI

Note: N and N events are not applicable for continuous variables

## Discussion

Our study examined the association between NAFLD and several sleep-related variables, including sleep duration and efficiency, wake-up and bed time, and variables indicative of poor sleep such as falling sleep unintentionally, having restless legs syndrome, and using sleeping pills, in a large cohort of ethnically-diverse middle-aged Iranian men and women. We found that a diagnosis of NAFLD with associated with worse sleep outcomes. Although part of these associations was explained by sociodemographic, lifestyle, or clinical variables, some remained strong after comprehensive confounder adjustment, in particular for sleep efficiency and use of sleeping pills, which suggests that NAFLD independently affects sleep. Across population subgroups, we found that: i) the association of NAFLD with sleep efficiency was stronger in older participants and those who were overweight / obese; and ii) the association of NAFLD with daytime napping was stronger in male participants and those with a BMI less than 30.

The strengths of our study were i) its large sample size, including 1,320 NAFLD cases drawn from a population-based cohort, ii) the clinical diagnosis of NAFLD made by ultrasonography; and iii) the adjustment for an extensive collection of sociodemographic, lifestyle, and clinical/biochemical variables. Our study also has several limitations. First, while ultrasonography and medical records were used to diagnose NAFLD, no liver biopsies could be obtained from the participants to improve diagnosis accuracy; liver biopsies are an invasive procedure associated with complications such as death and hemorrhage, so it was not a safe, feasible and cost-effective approach in this study. Patients with higher grades of NAFLD stated in their medical records and blood samples were referred to clinicians to take biopsy for cancer detection and excluded from the study. We also excluded participants with a history of excessive alcohol use to minimize the risk of NAFLD misclassification. The questionnaire we used to assess sleep characteristics was validated and used in several PERSIAN cohorts [20, 21], but other common sleep quality assessments such as the Pittsburgh Sleep Quality Index (PSQI) were not made. Like for all questionnaire-collected variables, the fact that sleep characteristics were self-reported may have resulted in measurement error, which usually yields estimates of association biased towards the null [27]. Due to its cross-sectional nature, our study did not allow conclusions about whether sleep disturbances were present before NAFLD development, or were caused by the presence of NAFLD; it nevertheless provides evidence that poor sleep is an important issue in NAFLD patients.

Our findings are partially in line with two recent cross-sectional studies [18, 28]. Kim et al. [18] found that NAFLD patients had shorter sleep duration in 45,293 middle-aged South Korean workers; the association was however only observed in the group with ≤ 5 h of sleep, compared with > 7 h and appeared stronger in women; men: odds ratio (OR) = 1.24 (95%CI: 1.11–1.39), women: OR = 1.55 (95%CI: 1.28–1.89), with effect modification by sex, P-interaction = 0.003. We found no effect modification by sex for sleep duration (P-interaction = 0.78) [18] and that short sleep duration was associated with only moderately increased NAFLD prevalence (+ 18% in age- and sex-adjusted models, and + 11% after comprehensive adjustments). Miyake et al. [28], in a community-based study of 2,429 Japanese participants aged 18–80 years, found a significant negative association between NAFLD and short sleep in men: adjusted OR = 0.55, 95%CI: 0.36–0.83, the same was not observed in women: OR = 0.97, 95%CI: 0.67–1.42. In contrast to our study this association was slightly increased in multivariable models after adjusting for age, SBP, TG, HDL, fasting plasma glucose, uric acid, ALT, creatinine, snacking habit, periodic exercise [28]. The meta-analysis by Wijarnpreecha et. al [9] included five cross-sectional and one cohort studies and participants with NAFLD were found to have shorter sleep duration: pooled RR: 1.19; 95%CI: 1.04–1.36. However, the meta-analysis of Shen et al. [12] did not find long sleep or short sleep to be associated with NAFLD. These findings taken together suggest that the association between sleep duration and NAFLD is relatively small. The choice of reference categories has also differed across studies which may complicate the interpretation of the findings. Additional studies are required to confirm whether this association varies by sex.

A common tool used to evaluate sleep quality in recent studies [18, 29, 30] was the global PSQI score of seven components including subjective sleep quality, sleep latency, sleep duration, habitual sleep efficiency, sleep disturbances, sleep medication use, and daytime dysfunction [31]. Our study used a different self-reported questionnaire, which was validated and detailed in a previous publication [20], and we evaluated each item individually. We found that participants with higher sleep efficiency were less frequently diagnosed with NAFLD, particularly in older participants and those with a higher BMI. A cross-sectional study of 4,828 Japanese participants found no association of NAFLD with global PSQI score and subjective sleep quality in both sexes, but similar to our results, NAFLD was associated with habitual sleep efficiency in women although this association did not remain after adjusting for BMI [29]. Chou et al. [30] examined the association of NAFLD with sleep quality using the global PSQI score in a study of 6,663 Taiwanese participants aged over 18 years. After adjusting for many confounders similar to our study, they found poor sleep quality to be associated with lower prevalence of both mild and moderate-to-severe NAFLD in men, but not in women [30]. Kim et al.’s study also examined the association between NAFLD and sleep quality [18]. A global PSQI score ≥ 5 (indicative of poor sleep quality) was positively associated with NAFLD in men after adjustment for sociodemographic, lifestyle and clinical variables [18]. The studies by Chou et. al [30] and Kim et. al [18] did not evaluate individual sleeping components separately as was done in Takahashi et al. [29] and our study.

Only few other studies have evaluated other sleep-related variables. Wang et al. [32] showed that bed time was associated with a diagnosis of NAFLD after adjusting for age, sex, and several metabolic variables in 22,807 Chinese participants; participants with later bed time were more likely to have NAFLD (compared with < 10 pm, 10 pm-12am: OR = 1.80 [1.05–1.32) and > 12am: OR = 1.42 [1.21–1.68)) [32]. This association was nevertheless substantially attenuated in comprehensively adjusted models in our study. In agreement with our findings, the authors found no interaction between BMI and bedtime [32]. A population-based study of 6,881 Chinese night-shift steelworkers suggested that the duration, length and frequency of night shift work were associated with NAFLD [33]. Late sleepers may be prone to metabolic disorders due to imbalances between external and internal clock, which can lead to postponing sleep and eating meals late at night, resulting in reducing melatonin secretion and disrupting circadian rhythm. Genetic studies have also found that genetic predispositions to late bed time and metabolic disorders were correlated [34–36].

Restless legs syndrome, one of the most common sleep disturbances in people with chronic conditions [37, 38], was negatively associated with NAFLD in our study, although the association was attenuated in comprehensively-adjusted models (RR = 0.87, 95% CI: 0.73–1.04). Mir et al.’s population-based study of 10,541 in the US found a weakly positive association [39]. Similarly, for daytime napping, our results were partially in agreement with two cross-sectional studies [17, 40]. A community-based population study of 8,559 middle-aged and elderly Chinese participants found that those with long nap duration (> 0.5 h) (OR = 1.22, 95% CI: 1.06–1.41) were more likely to have NALFD compared with those who did not nap; the authors did not find effect modification by obesity status as in our study, but findings are difficult to compare because of different BMI cut-off points used, and a lower proportion of obese participants in the Chinese study [17]. Another cross-sectional study of middle-aged Chinese participants suggested that NALFD was more frequent in participants reporting > 0.5 h napping per day compared with no napping [40]. Studies taken together are consistent with a weak association between daytime napping and NAFLD.

Finally, the use of sleeping pills was substantially more frequent (+ 50%) in our participants with NAFLD, which likely reflected their history of poor quality / short sleep or medical conditions. This association was i) robust to comprehensive adjustments and ii) consistent across age, sex, and BMI subgroups. Since sleeping pills improve sleep quality and extend sleeping time, this better regulation of the circadian rhythm could theoretically be beneficial for patients with metabolic conditions via an effect on glucose metabolism and insulin sensitivity [41]. Such beneficial effect in reducing the risk of NAFLD is nevertheless unclear and should be examined in prospective studies.

## Conclusions

Poor sleep quality is a common issue in middle-aged Iranians with NAFLD, and the associations we observed were not fully explained by sociodemographic, lifestyle, or other clinical conditions. Although our study could not unravel the causal relationship between quality and duration of sleep and NAFLD, it highlights that sleep disturbances may affect the quality of life of these patients, and potentially worsen their prognosis.

## Data Availability

The SCS data are not open-access, but external researchers can use the data for collaborative projects. The process relative to data access and collaboration can be obtained from the corresponding author Dr. Ali Ahmadi, or at info@ persiancohort.com.

## Abbreviations

NAFLD: Non-alcoholic fatty liver disease
PERSIAN: Prospective Epidemiological Research Studies in Iran
BMI: Body mass index
MetS: Metabolic syndrome
T2D: Type 2 diabetes
SCS: Shahrekord Cohort Study
HDL: High-density lipoprotein
LDL: Low-density lipoprotein
ALT: Alanine aminotransferase
AST: Aspartate aminotransferase
TG: Triglycerides
WC: Waist circumference
SBP: Systolic blood pressure
DBP: Diastolic blood pressure
SD: Standard deviation
RR: Relative risks
CI: Confidence intervals
ATPIII: National Cholesterol Education Program Adult Treatment Panel III criteria
PSQI: The Pittsburgh Sleep Quality Index
OR: odds ratio
